# PCR with electrospray ionization-mass spectrometry on bronchoalveolar lavage for detection of invasive mold infections in hematological patients

**DOI:** 10.1371/journal.pone.0212812

**Published:** 2019-02-22

**Authors:** Anders Krifors, Volkan Özenci, Måns Ullberg, Malin Ackefors, Martin Jädersten, Kristoffer Strålin, Ola Blennow

**Affiliations:** 1 Department of Physiology and Pharmacology, Karolinska Institutet, Stockholm, Sweden; 2 Department of Clinical Microbiology, Karolinska University Hospital, Stockholm, Sweden; 3 Division of Clinical Microbiology, Department of Laboratory Medicine, Karolinska Institutet, Stockholm, Sweden; 4 Department of Infectious Diseases, Karolinska University Hospital, Huddinge, Sweden; 5 Centre for Haematology and Regenerative Medicine, Department of Medicine Huddinge, Karolinska Institutet, Stockholm, Sweden; 6 Department of Medicine Huddinge, Karolinska Institutet, Stockholm, Sweden; Wadsworth Center, UNITED STATES

## Abstract

Invasive mold infections are life-threatening complications in patients with hematological malignancies. Conventional microbiological methods for diagnosing invasive pulmonary mold infections have low sensitivity, and molecular methods are being developed. Detection of molds using PCR with a narrow spectrum has been reported, but data with broad-spectrum PCR are lacking. In this study, the diagnostic performance and utility of a broad-spectrum PCR (broad-spectrum PCR with subsequent electrospray ionization-mass spectrometry, PCR/ESI-MS) for detection of molds in bronchoalveolar lavage (BAL) in 27 hematological patients with a new pulmonary infiltrate was analyzed. Using the revised EORTC/MSG criteria, PCR/ESI-MS was the only positive microbiological test in patients with proven invasive mold infection (n = 2) and correctly identified all cases of probable invasive pulmonary aspergillosis (n = 5). In patients with a possible invasive mold infection (n = 5), PCR/ESI-MS was positive in three patients. *Mucorales* was identified with PCR/ESI-MS in four patients that were all culture negative. The PCR/ESI-MS results had a clinical impact on antifungal therapy in 12 (44%) of the patients: modification of treatment in 6 (22%) patients and discontinuation in 6 (22%) patients. This study provides proof of concept that routine use of a broad-spectrum PCR for molds in bronchoalveolar lavage in immunocompromised patients is sensitive, fast, and has an impact on clinical decision-making

## Introduction

Invasive mold infections are life-threatening complications in patients with hematological malignancies, and adequate early treatment has been shown to be essential for a successful outcome [1,2]. Identification of non-*aspergillus* molds, especially molds from the order *Mucorales* causing mucormycosis, is of particular concern because of their intrinsic resistance to antifungal agents and the often aggressive course of the infections [3,4]. Conventional microbiological methods for detection of molds, i.e. microscopy and culture, are limited by low sensitivity and unacceptable long turn-around time and more sensitive tests targeting biomarkers have been developed. However, of the tests most commonly used today, galactomannan (GM) and Aspergillus PCR can only detect *Aspergillus spp*. and β-D-Glucan, although having a broader mold spectrum, cannot detect *Mucorales* [5]. Thus, new broad-spectrum and molecular-based, diagnostic tests are much needed. One such test is broad-spectrum PCR with subsequent electrospray ionization-mass spectrometry (PCR/ESI-MS) which can identify more than 200 fungal species with a turnaround time of under 7 hours [6–10]. Between February 2016 and May 2017, PCR/ESI-MS was available as a routine diagnostic method at Karolinska University Hospital, as one of only two hospitals worldwide. In the present study, the diagnostic performance and clinical utility of routine use of a broad-spectrum PCR for detection of molds in bronchoalveolar lavage (BAL) in hematological patients with a new pulmonary infiltrate was evaluated. We hypothesized that PCR/ESI-MS would be a sensitive and fast technique that would also have an impact on clinical decision making.

## Material and methods

This retrospective cohort study includes all hematological patients at Karolinska University Hospital Huddinge Stockholm, Sweden, that had a diagnostic bronchoscopy performed between February 2016 and May 2017 due to a new pulmonary infiltrate diagnosed by computer tomography. During this period PCR/ESI-MS was included as an optional routine test when a diagnostic BAL was performed. If a patient had more than one BAL performed, only the results from the first BAL were included. Low volume BAL was performed using a standard protocol of 10–20 mL of 0.9% saline solution inserted and subsequently retrieved for analysis. Microscopy, bacterial and fungal cultures, and GM were performed in all patients while other diagnostic tests were ordered per the discretion of the referring physician. All microbiological analyses presented were conducted at the Department of Clinical Microbiology, Karolinska University Laboratory, Karolinska University Hospital, Stockholm. GM testing was performed using the Bio-Rad Platelia kit per manufacturers´ instructions. A positive cut-off level of 0.5 for serum and 1.0 for BAL fluid was used per the manufacturers' recommendation. Aspergillus PCR was performed using a method compliant with the European Aspergillus PCR initiative guidelines [11,12]. The method is a qualitative DNA detection by amplification of a 483–505 bp fragment (depending on the pathogen) of the 18S rRNA gene of *Candida* and *Aspergillus spp*. with singleplex real-time PCR. Hybridization probes labeled with LC670 and LC640, respectively, allow PCR product detection and subsequent melting curve analyses.

Broad-spectrum PCR was performed using a PCR/ESI-MS assay (IRIDICA Fungal Assay, Ibis Biosciences, Abbott, Des Plaines, USA) according to the manufacturer's instructions. From each sample, an aliquot of 5 ml BAL was analyzed. Samples were processed according to the manufacturer’s recommendation, including mechanical and chemical lysis; extraction of DNA followed by PCR; desalting and purification of the amplicons; and analysis by ESI-MS. Species identification was achieved by bioinformatical analysis with integrated software and database. Quantitation of detected genomes was based on internal calibrant molecules in each reaction and was reported as a semi-quantitative “level” for each detection.

All microbiology findings of yeast were regarded as clinically insignificant and not included in the analyses. Similarly, molds found in cultures and/or broad-spectrum PCR that were regarded to be contaminants or colonizers according to local guidelines were not included in the analyses.

The revised 2008 EORTC/MSG criteria for the definition of invasive fungal disease were used when evaluating the diagnostic performance of PCR/ESI-MS compared to other diagnostic tests [13]. Following the 2008 EORTC/MSG criteria, Aspergillus PCR was not included as mycological criteria. Clinical data were collected retrospectively from medical records.

Ethical permit was obtained by The Regional Ethics Committee in Stockholm (approval number, EPN 2016-2253-31-1). The ethics committee waived the requirement for informed consent to retrieve data from the patients' medical records. After retrieval, all data were fully anonymized.

## Results

**Fig 1:**
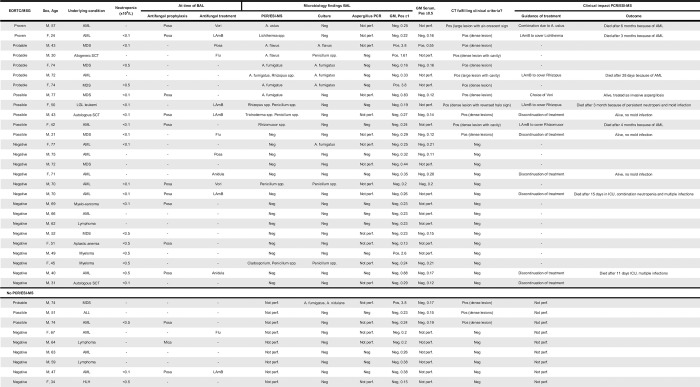
Patients characteristics and results.

In total, 36 patients had at least one BAL performed during the study period. The most common underlying hematological diseases were acute myeloid leukemia (n = 15) and myelodysplastic syndrome (n = 8) (Fig 1). The median age was 61 years (range 21–77). During the week preceding the bronchoscopy, 22 patients (61%) an absolute neutrophil count of <0.5 x 10^9^/L at least once, of which 13 patients (36% of all patients) had <0.1 x 10^9^. Seventeen patients (47%) received mold-active prophylaxis or empiric treatment at the time of BAL (Fig 1). Microscopy, fungal culture, and GM were performed on all BAL samples (n = 36), PCR/ESI-MS PCR was performed on 75% (n = 27), and Aspergillus PCR on 47% (n = 17) of the BAL samples. Serum GM was performed in 47% (n = 17) of the cases (Fig 1). There was no significant difference in proven or probable invasive mold infections between patients in whom PCR/ESI-MS was performed (7/27; 26%) and not performed (1/9;11%; p = 0.65, Fischer´s exact test).

**Fig 2:**
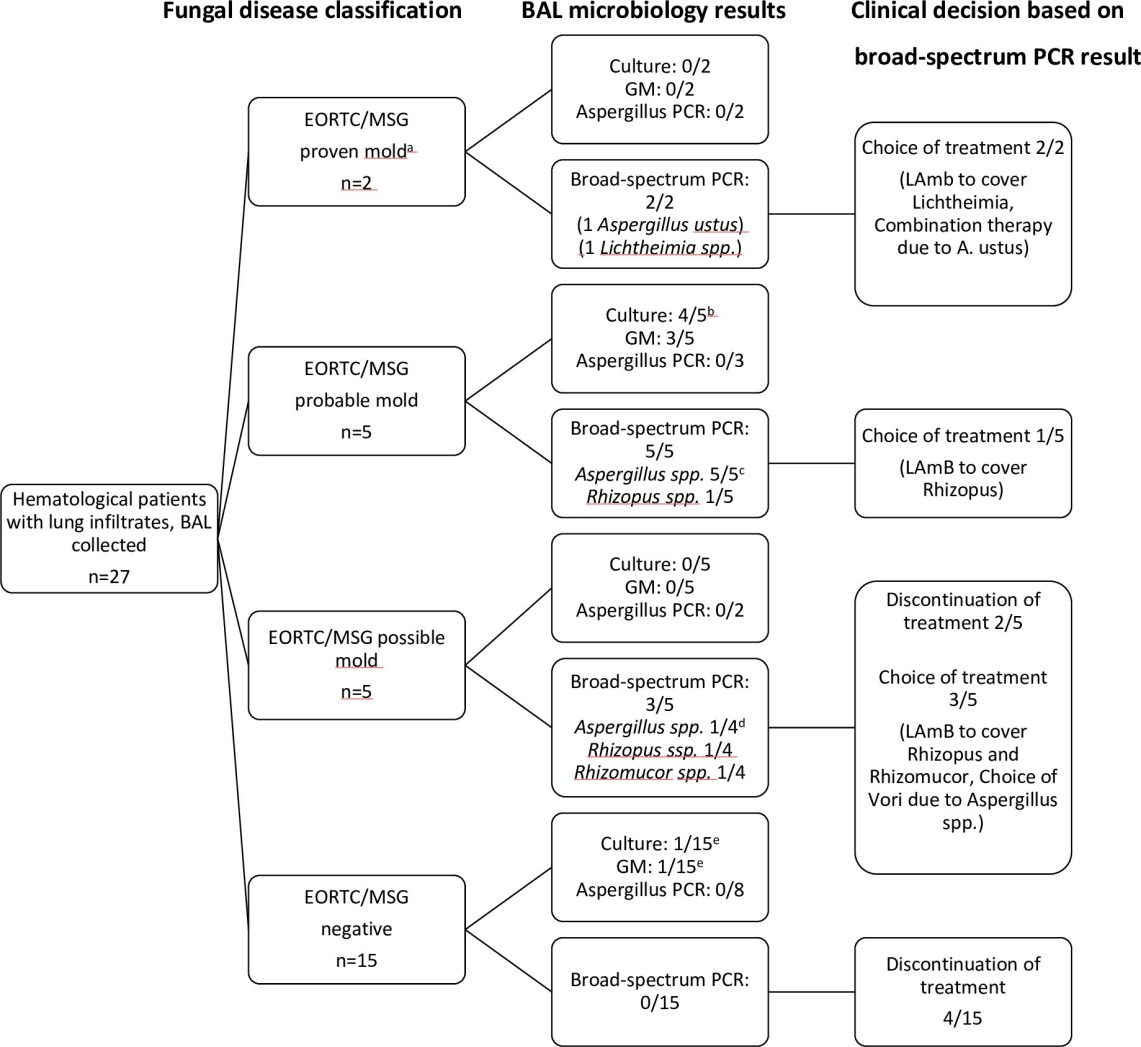
Microbiology findings in BAL where PCR/ESI-MS was performed. *BAL*: *bronchoalveolar lavage; GM*: *galactomannan*. ^a^Microscopic findings of hyphae in lung biopsy in both patients. ^*b*^*Aspergillus fumigatus*: 3, *Aspergillus flavus*: 1. ^c^*Aspergillus fumigatus*: 2, *Aspergillus fumigatus+Rhizopus spp*: 1, *Aspergillus flavus*: 2. ^*d*^*Aspergillus fumigatus*. ^e^Growth of *Aspergillus fumigatus* in one patient and GM positive in another patient, but the pulmonary infiltrates did not meet clinical criteria in either patient. Both were treated as invasive pulmonary aspergillosis.

The diagnostic yield and clinical impact of PCR/ESI-MS (n = 27) are presented in (Fig 2). In two patients with proven invasive mold infections, PCR/ESI-MS was the only positive microbiological test in BAL. One patient developed an infiltrate with a crescent sign while receiving posaconazole prophylaxis. PCR/ESI-MS was positive for *Aspergillus ustus* and treatment was switched to voriconazole. Due to treatment failure, a lung biopsy was performed after one month of treatment with findings of hyphae but the culture was negative. An echinocandin was added to the ongoing voriconazole treatment resulting in a slight regression of the infiltrate after one month. The patient died later due to the underlying hematological disease while receiving posaconazole. The other patient had a relapse of AML after allogeneic stem cell transplantation and developed a large, dense, infiltrate with necrosis while receiving posaconazole. PCR/ESI-MS was positive for *Lichtheimia spp*. and liposomal amphotericin B (LAmB) was started leading to regression of the infiltrate. Due to the size of the infection, a lobectomy had to be performed, and microscopy showed findings suspicious of *Mucorales*.

PCR/ESI-MS was able to detect *Aspergillus* in all patients (n = 5) with probable invasive aspergillosis (IA), diagnosed by positive culture (n = 4) and/or positive GM test (n = 3). In one patient with growth of *Aspergillus fumigatus*, but negative GM, PCR/ESI-MS was positive for both *A*. *fumigatus* and *Rhizopus spp*. CT showed a dense infiltrate with necrosis but with neither a halo nor reversed halo sign. A polymicrobial infection could not be ruled out and led to the initiation of treatment with LAmB. The invasive mold infection in combination with the patient´s age and underlying condition led to transition to palliative care making it impossible to evaluate the treatment effect of LAmB.

Excluding the two patients with proven infection but of unknown etiologies, the sensitivity and specificity of PCR/ESI-MS for probable invasive lung aspergillosis was 100% (5/5) and 95% (19/20) respectively. This corresponds to a positive predictive value of 83% and a negative predictive value of 100%.

In patients with possible invasive mold infection (n = 5), PCR/ESI-MS was positive in three patients with findings of *A*. *fumigatus*, *Rhizopus spp*., and *Rhizomucor spp*. These were all considered to be significant and treated accordingly. Three of the five patients with possible mold infection had received mold-active prophylaxis or treatment before BAL which might have contributed to the negative cultures and GM tests. The patient positive for *A*. *fumigatus* had been neutropenic for 30 days without mold-active prophylaxis and had a fever and an elevated CRP-level despite broad-spectrum antibiotics. CT showed multiple dense lesions with necrosis, the largest over 3 cm in diameter. Voriconazole was started leading to clinical improvement and regress of infiltrates on CT. The patient with *Rhizopus spp*. had been neutropenic for three months when CT showed a dense infiltrate with necrosis. The initial BAL was culture negative and GM negative (PCR/ESI-MS not performed) and LAmB was started. A new BAL was performed after 14 days of treatment with the finding of *Rhizopus spp*. in PCR/ESI-MS. The patient had persistent neutropenia and died after three months of treatment due to a progression of the mold infection. The third patient was receiving prophylaxis with posaconazole because of neutropenia while waiting for allogeneic stem cell transplantation. CT showed an infiltrate with inversed halo sign and PCR/ESI-MS was positive for *Rhizomucor spp*. LAmB was started but transplantation had to be canceled leading to a transition to palliative care.

In total, PCR/ESI-MS results had an influence on the antifungal therapy in 12 of 27 patients (44%) (Fig 2). In six patients the negative results influenced the decision to discontinue treatment. Four of these patients survived with no clinical diagnose of invasive mold infection, while two patients died due to the underlying hematological disease in combination with infectious complications where mold infections could neither be ruled in or out (Fig 1). In six patients the PCR/ESI-MS results led to either initiation of antifungal treatment or changes in the ongoing therapy. In the four patients with PCR/ESI-MS positive for molds belonging to *Mucorales*, the results led to a change in treatment from an azole to LAmB (Fig 1 and Fig 2).

Molds considered to be contaminants or colonizers according to local guidelines were found in 4/27 (15%) patients with broad-spectrum PCR (*Penicillium spp*. n = 4, *Trichoderma spp*. n = 1, *Cladosporium spp*. n = 1) and in 3/36 (8%) patients with culture (*Penicillium spp*. n = 3). In two patients, PCR/ESI-MS and culture were concordant.

## Discussion

In this retrospective cohort study, the routine use of PCR/ESI-MS on BAL in hematological patients was found to have an impact on antifungal treatment in 44% of patients. Because of the low sensitivity of conventional microbiological tests focus has shifted to PCR-based techniques for identifications of non-*Aspergillus* molds. Several studies reporting successful development of non-*Aspergillus* PCR have been published, but the tests have suffered the disadvantage of being in-house analyses and/or having a narrow diagnostic spectrum [14]. In the present study, use of a standardized broad-spectrum PCR was able to accurately identify all five cases of probable IA, as well as four otherwise undetected cases of non-*Aspergillus* molds belonging to the order *Mucorales*. An important advantage of PCR-based methods compared to culture-based methods is the potential to identify molds after empirical antifungal therapy has been initiated, a situation which is common in clinical practice. Interestingly, PCR/ESI-MS could identify clinically important molds in three of the five patients that were already receiving mold-active treatment and had lung filtrates compatible with an invasive mold infection.

Besides detecting invasive mold infections, it is also important to identify true negative results, allowing for discontinuation of antifungal treatment. In this study a negative PCR/ESI-MS result was an important factor in the decision to discontinue antifungal treatment in six (22%) of the patients, thus avoiding both potential side effects and high costs. Another advantage was a fast turnaround time with a result reached the same or following day after sampling.

There were some potential limitations in the diagnostic strategy for IA used during the study period. The diagnostic approach to pulmonary infiltrates in hematological patients consisted of performing BAL for culture, GM, and Aspergillus PCR. Since both GM and Aspergillus PCR have been shown to have higher sensitivity in BAL than in blood for diagnosing invasive pulmonary aspergillosis, Aspergillus PCR analyses in blood were not performed at all. The diagnostic approach to pulmonary infiltrates in hematological patients consisted of performing BAL for culture, GM and Aspergillus PCR. Since both GM and Aspergillus PCR have been shown to have higher sensitivity in BAL than in blood for diagnosing invasive pulmonary aspergillosis, Aspergillus PCR in blood was not performed while GM in blood was performed according to the discretion of the treating physician [15–19]. β-D-Glucan was not used because the test was not available as a routine analysis during the study period. Combining non-culture based diagnostic tests in BAL such as GM and Aspergillus PCR in BAL has been shown to increase the sensitivity for IA [20,21]. Unfortunately, Aspergillus PCR was performed in only half of the patients during the study period, probably because PCR/ESI-MS was considered to be a replacement for Aspergillus PCR instead of being a compliment. In patients that Aspergillus PCR was performed, the test was negative in four patients with probable IA. The in-house PCR method that was used in this study has been in clinical routine since 2002. Clinical results using the method have been published and the results in that study and in current clinical routine show that the false negative results by our in-house PCR are low [22]. It is thus difficult to understand the reason for these four false negative results. It cannot be ruled out that that more cases of IA would have been identified if another Aspergillus PCR method had been used and if Aspergillus PCR had been analysed in all BAL samples, but that would not change the classification of cases as PCR is not a part of the EORTC/MSG criteria. Routine use of β-D-Glucan might have had an impact on the five possible cases, changing the classification to probable infection, but likely in favor of PCR/ESI-MS as one patient was positive for *A*. *fumigatus* in PCR/ESI-MS and was clinically considered to be a true infection.

Another limitation of the study is that not all BAL performed during the study period were subjected to PCR/ESI-MS testing. However, the proportion of proven or probable infections was comparable among the two groups, indicating that the absence of PCR/ESI-MS testing was a random event.

Low volume BAL was used to minimize respiratory distress, and the results may not be applicable for regular BAL. One strength of the study is that it was conducted during a short period and in a single center setting, without changes in treatment protocols or prophylaxis during the study period.

In summary, PCR/ESI-MS on low volume BAL in hematological patients with a new pulmonary infiltrate yielded valuable microbiological information and had a substantial impact on antifungal therapy. Unfortunately, PCR/ESI-MS was discontinued for commercial use by Abbot Laboratories in April 2017 [23]. The present encouraging data brings new insight into the importance of broad panel molecular-based methods in the detection of molds and will hopefully inspire manufacturers to pursue similar methods with high sensitivity and specificity.

## Supporting information

S1 Table Primers and probes included in real-time PCR (18S rRNA)for detection of *Candida spp*. and *Aspergillus spp*. (TIF)
