# Supplementary figures and images for: PCR with electrospray ionization-mass spectrometry on bronchoalveolar lavage for detection of invasive mold infections in hematological patients
Source: PLoS One. 2019 Feb 22;14(2):e0212812. doi: 10.1371/journal.pone.0212812 (PMC6386253; doi:10.1371/journal.pone.0212812)

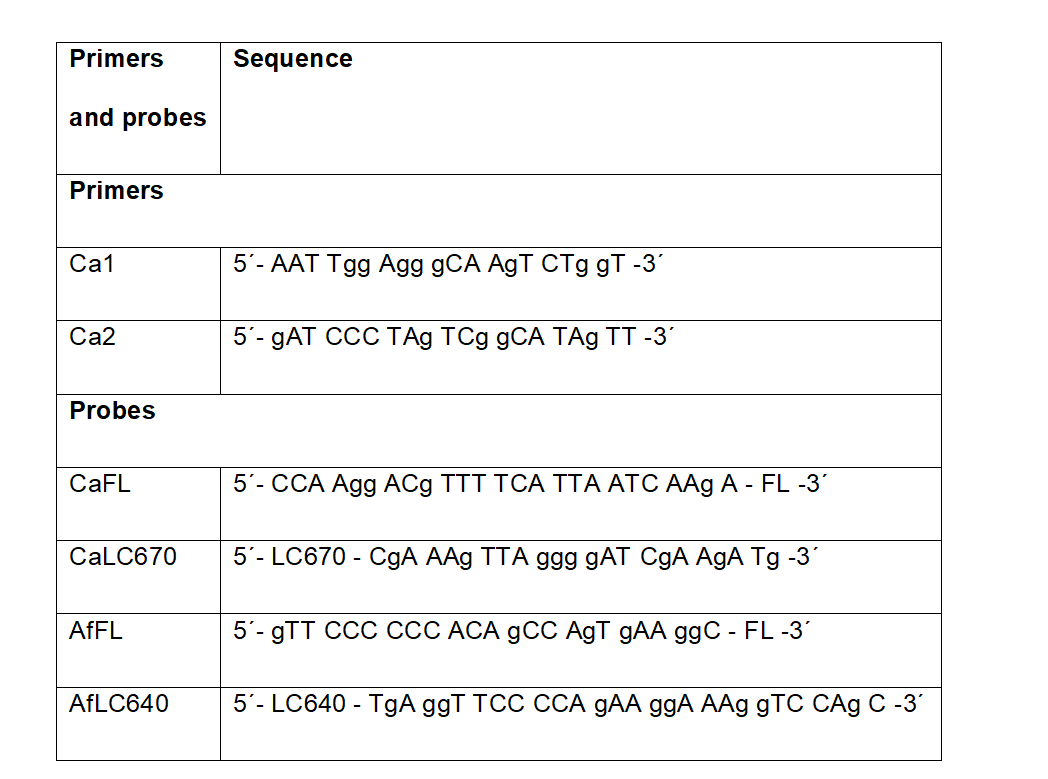

Supplement: S1 Table — (TIF) [file pone.0212812.s001.tif]
